# Young Australian Adults Prefer Video Posts for Dissemination of Nutritional Information over the Social Media Platform Instagram: A Pilot Cross-Sectional Survey

**DOI:** 10.3390/nu14204382

**Published:** 2022-10-19

**Authors:** Virginia Chan, Margaret Allman-Farinelli

**Affiliations:** 1 Discipline of Nutrition and Dietetics, Susan Wakil School of Nursing and Midwifery, Faculty of Medicine and Health, The University of Sydney, Sydney, NSW 2006, Australia; 2 Charles Perkins Centre, The University of Sydney, Sydney, NSW 2006, Australia

**Keywords:** Instagram, social media, young adults, nutrition, health promotion

## Abstract

Growing social media use in young adults may have applications in health promotion. This study aimed to determine the acceptability and feasibility of using Instagram to disseminate nutritional information to young Australians and assess the most preferred post style. A cross-sectional web-based pilot survey was conducted in 18–30-year-olds residing in New South Wales. Eight sets of mock Instagram posts were generated comprising three formats: (i) text/icon, (ii) realistic image, or (iii) video. Respondents (*n* = 108) were asked to review and rank posts from highest to lowest according to likelihood of engagement, visual preference, motivation to change eating behaviors, and relevancy of information. The Friedman test (Wilcoxon signed-rank test post hoc analysis with Bonferroni correction) was conducted to determine differences between the three post styles. Video style posts were more likely to be engaged with (*p* < 0.001), visually preferred (*p* < 0.001), more motivating to change eating behaviors (*p* < 0.001), and presented the most relevant food and nutrition knowledge (*p* < 0.001) compared with the other post styles. Most participants reported that Instagram was a suitable platform to share food and nutrition information (96%). The findings of this pilot study can be used to inform a large study that investigates the use of Instagram among a more diverse population and with a greater number of video posts tailored for audience segmentation.

## 1. Introduction

Young Australian adults are more likely to gain weight than all other age groups [1]. The proportion of young people affected by overweight and obesity increased from 39% in 2014–2015 to 46% in 2017–2018 in the Australian National Health Survey [2]. Diet is one major modifiable factor against the global obesity pandemic.

Young adults have been previously found to have poor diet quality [3]. Snacks are of particular concern as they are an important contributor to total energy intake. Snacks are foods consumed outside the three main meals: breakfast, lunch, and dinner. Secondary analysis of the Australian National Nutrition and Physical Activity Survey (NNPAS) 2011–2012 identified that snacks contributed to 20% of the total energy intake in adults (>19 years) [4], and frequency of snacking was associated with overweight and obesity [5]. A large proportion of Australians aged 19–30 years (88%) reported snacking, with 41% of energy intake from snacks coming from discretionary items [4]. Discretionary foods are defined in the Australian Guide to Healthy Eating (AGHE) as foods that are high in saturated fat, and/or sugars, and/or salt, and/or low in fiber [6].

Another important contributor to energy intake is food prepared outside the home. The Measuring Young Adult Meals Study (MYMeals) was a large cross-sectional study of 1001 Australians aged 18–30 years old. This study was conducted in 2017–2018 and aimed to measure the contribution of food prepared outside the home to total energy and nutrients of concern. This study found that one third of the food and beverages consumed was prepared outside the home but proportionally had more energy, nutrients, and sodium [7]. Analysis of a subsample of the MYMeals study identified that food prepared outside the home environment was more likely to consist of “unhealthy” discretionary items than food prepared within the home [8]. It was also found that food prepared outside the home and snacks in particular were more energy-dense than those prepared within the home and all other meal types [9].

Modifying dietary behaviors is a potential avenue for public health interventions. However, young adult populations are difficult to reach due to their low healthcare utilization rate [10]. Only 71.2% of young people (15–24 years) engaged with a general practitioner compared to 94.8% for older individuals (>85 years), making it difficult to disseminate information to this age group [11]. Social media offers a unique opportunity for dietary health promotion, as young people are frequent uses of these platforms. Almost 90% of a sample of young adults accessed social media at least once per day and 55% reported checking ten or more times each day [12]. The majority of studies examining the use of social media for nutrition health promotion have been conducted using Facebook, Twitter, or custom-designed platforms [13]. However, it has been reported that young adults are moving away from these platforms towards more image- and video-based content such as Instagram [14].

The use of Instagram in 18–29-year-old Australians has increased from 58% in 2016 [12] to 81% in 2017 [15]. The rising popularity of Instagram offers a new opportunity to deliver health promotion programs at a relatively low cost. A survey conducted in Brazilian dietitians identified that Instagram was the most popular social networking site and was commonly used to generate content related to food and nutrition [16]. Instagram has been previously used to implement a social media campaign promotion for healthy eating during the COVID-19 pandemic in Canada [17] and delivered an exercise program in Australian females aged 18–30 years [18].

Therefore, the aim of this study was to determine the acceptability and feasibility of using Instagram to disseminate nutritional information to young Australian adults. This study also aimed to determine which post styles (text/icon, realistic images, or videos) respondents rated highest in four domains (engagement, visual preference, motivation to change eating behaviors, and relevancy of information).

## 2. Materials and Methods

### 2.1. Study Design

A cross-sectional web-based survey was conducted in young Australian adults (18–30 years) to understand their perceptions of using the social media platform Instagram to share nutritional information. All materials and methods for the online survey were approved by the Institution’s Human Research Ethics Committee (2022/522) on the 25 July 2022. The CROSS (Consensus-Based Checklist for Reporting of Survey Studies) [19] framework was used to direct the reporting of the survey results.

### 2.2. Content Development

**Figure 1:**
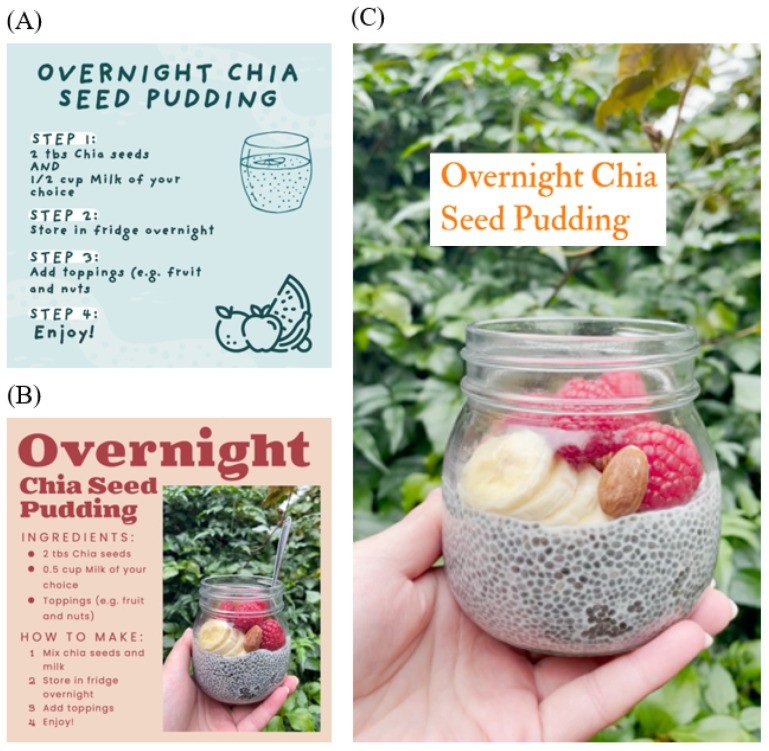
Sample set of three mock Instagram social media posts for a chia seed pudding recipe in the following formats: (**A**) text and icons only; (**B**) realistic image; and (**C**) short video.

Twenty-four (eight sets of three) mock Instagram social media posts were generated by an accredited practising dietitian (APD) (V.C.). The content of these posts was guided by the key findings of the wearable camera MYMeals sub-study as well as the larger study of 1001 young adults to ensure that the observed needs of this population were targeted, which are, in summary, to assist food choice when consuming food prepared outside the home and to consume fewer energy-dense snacks. The posts covered: (i) snacking behaviors (four sets of three posts) [9] and (ii) consuming food prepared outside the home (four sets of three posts) [8,9]. However, one of the key objectives of this study was to examine the preferred delivery vehicle of nutritional information. Thus, each set of posts consisted of three separate individual posts that conveyed the same information in the following formats: (i) text and icons only, (ii) realistic images, or (iii) short videos (Figure 1).

The Adobe Express Online Application (Adobe Inc., San Jose, CA, USA, https://express.adobe.com/sp/) was used to generate the: (i) text and icon only, (ii) realistic image, and (iii) the onscreen captions for the video posts. All realistic images were captured using an Apple iPhone 13 smartphone or used the Adobe (royalty-free stock) images offered through the Adobe Express Online Application.

The short videos were captured on an Apple iPhone 13 smartphone that was either: (i) attached to a tripod using a smartphone mount or (ii) held freehand. Video footage was imported into Adobe Rush version 2.3.0 (Adobe Inc., San Jose, CA, USA), which was used to: (i) edit the videos; (ii) add onscreen captions and/or images; (iii) insert background (royalty-free stock) music; and (iv) insert voiceovers recorded within the software using an external microphone (Rode Wireless Go 2, Rode, Sydney, Australia).

### 2.3. Participant Eligibility Criteria

Prospective participants were eligible to take part in the study if they were within the age range (18–30 years old), resided in New South Wales (NSW), and used Instagram. Participants were excluded if they did not meet the eligibility criteria or were pregnant and/or breastfeeding.

### 2.4. Recruitment Methods

Participant recruitment commenced on 10 August 2022 and proceeded until the calculated sample size was achieved. Sample size was determined using the Australian Bureau of Statistics’ online Sample Size Calculator tool [20]. This online tool calculates the required sample size by inputting the confidence interval, either the standard error or relative standard error, and population, which was estimated as 70,000 [20]. The parameters of a 95% confidence interval and a 10% relative standard error yielded a target sample size of 100.

Convenience sampling was used to recruit participants. Recruitment methods included posting the recruitment flyer on social media platforms (Instagram, Facebook, Reddit, and Discord, no paid targeted advertisements were used) and on public noticeboards on one university campus where 70,000 students attended. The recruitment flyer was also posted on student newsletters and university online learning management systems via university staff, and the study information was advertised on the university volunteer for research webpage at the same campus. In the recruitment information, the study was described as an online survey that aimed to explore different post styles that could be used to share nutritional information on Instagram.

### 2.5. Study Procedure

Those interested in participating could scan the Quick Response (QR) code or use the weblink on the recruitment poster to access the online initial screening questionnaire. If participants were eligible, they were provided with the Participant Information Statement and Participant Consent Form. Those who provided informed consent were taken to the demographics questionnaire. The demographics questions included: (i) gender (male, female, other, prefer not to say); (ii) age range (18–24 years or 25–30 years); (iii) residential postcode; and (iv) level of education (currently enrolled or graduated from a university degree, currently enrolled or graduated from a trade qualification, apprenticeship certificate or diploma (TAFE or business college), school (only), or prefer not to say).

This was followed by the main study questionnaire that consisted of ten main questions. These asked the participants about their current use of Instagram and to review and rank the mock posts according to which style they: (i) were most likely to engage with (like, save, or share); (ii) visually preferred; (iii) experienced increased motivation to change eating behaviors; and (iv) believed displayed the most relevant food or nutrition knowledge. Finally, participants were asked if they considered Instagram to be an appropriate social media platform to share nutrition information. Participants were required to provide their email to receive an AUD 10 voucher in compensation for their time upon completing the study. The questionnaire is available upon request from the corresponding author.

The survey was closed when the target sample size of 100 was achieved. Participant emails were checked for duplicates upon conclusion of the data collection to prevent multiple participations of participants.

All participant data was de-identified, captured, and stored on REDCap electronic capture tool version 12.0.7 hosted at the University [21,22]. REDCap is a secure web application to build and manage online surveys. Access to the data was restricted to select members of the research team.

### 2.6. Data Analysis

Self-reported postcodes and the socio-economic indexes for areas (SEIFA) [23] were used to assign participants decile for advantage versus disadvantage, with lower deciles demonstrating more disadvantage. In the rare event that the participant’s residential postcode did not have an associated SEIFA value (*n* = 10), SES was assigned based on the SEIFA decile of the adjacent postcode area. The participants’ socioeconomic statuses (SES) were reduced to two categories: lower SES (the bottom five SEIFA deciles) and higher SES (the top five SEIFA deciles) due to the small sample size in some cells. Participants with an invalid NSW postcode were removed from SES reporting but included in the final analysis.

Descriptive statistics (frequency (*n*) and percentages (%)) were used to determine sample characteristics, participant Instagram usage, and the reported appropriateness of Instagram to share nutrition information. Participants responses towards the posts were tabulated as follows: (i) the lowest ranked post was allocated a value of 1; (ii) the neutral ranked post was allocated a value of 2; and (iii) the highest ranked post was allocated a value of 3. The median rank (and interquartile range (IQR)) were calculated per post style for engagement, visual preference, motivation, and relevancy of food or nutrition knowledge. There were no missing data; the survey was established such that respondents were required to provide an answer to all questions before they could proceed to the subsequent question.

A chi-square test was used to determine any differences in demographics (age group) between completers and non-completers (those that provided consent but did not complete the main study questions). No analysis on the impact of recruitment method on results was completed, as information was not collected in the survey.

The Friedman test was conducted to determine if there was a difference between median ranks and post style using SPSS software, version 25.0 for Windows (IBM Corp, Armonk, NY, USA) with a significance level of 0.05. If significant, a post hoc analysis with the Wilcoxon signed-rank test was conducted with a Bonferroni correction applied to determine differences between the three post styles (significance level was 0.017).

Mann–Whitney U tests were conducted to examine differences by participant demographics (gender, age range, education, SES, and frequency of Instagram use) for median post rank according to the post style with a Bonferroni correction applied (significance level was 0.017). The analysis excluded those who responded “prefer not to say” and “other” for gender and those that did not report a valid NSW postcode. Due to small numbers in some cells, education was reduced to: “currently enrolled or graduated from a university degree” and “all other education”. The “all other education” encompassed participants who reported their education to be “currently enrolled or graduated from a trade qualification, apprenticeship certificate or diploma (TAFE or business)”, “school (only)” or “prefer not to say”. Frequency of Instagram use was also reduced to: “multiple times per day” and “once per day or less”. The “once per day or less” group encompassed participants who reported to use Instagram “once per day”, “a few times per week”, or “less than once per week”.

## 3. Results

### 3.1. Sample Characteristics

**Figure 2:**
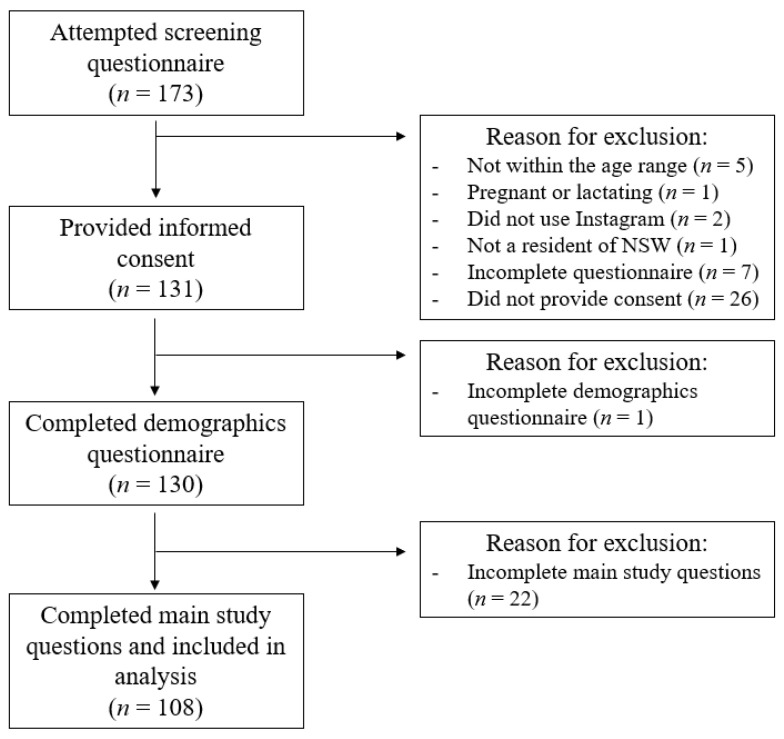
Participant flow diagram in the Instagram survey study.

Data were collected over three days (10–12 August 2022) and the survey closed after 100 complete responses were returned. The initial screening questions were attempted by 173 participants, and 108 respondents completed the main study questions (62%) and were included in analysis. Participants were excluded if they were not within the 18–30 age range (*n* = 5), were pregnant or lactating (*n* = 1), did not use the social media platform Instagram (*n* = 2), were not a resident of NSW (*n* = 1), did not complete the screening questionnaire (*n* = 7), did not provide consent (*n* = 26), or did not compete the demographics (*n* = 1) or main study questions (*n* = 22). A flow of participants through the study is shown in Figure 2.

The participant demographics are shown in Table 1. The sample comprised mostly females (77%), participants within the 25–30 age range (55%), participants currently enrolled in or graduated from a university degree (68%), and participants from a higher SES (89%). Most respondents reported checking Instagram at least once daily (74%) with 51% of participants reporting checking the app multiple times daily. Ten participants reported postcodes that did not have a designated SEIFA [23], but they were assigned to the SEIFA for adjacent postcodes (*n* = 10 higher SES).

There was no difference between the age groups (*p* = 0.691) of completers and non-completers of the main study. Due to the small sample size in some cells, differences between completers and non-completers of the main study could not be calculated for gender and SES.

### 3.2. Acceptability and Feasibility of Instagram to Share Food and Nutrition Information

Most participants responded that Instagram was a suitable platform to share food and nutrition information (*n* = 104, 96%). The four participants that responded that Instagram was not suitable nominated TikTok (*n* = 1) and Reddit (*n* = 1) as potential platforms, and two participants reported social media was not a suitable medium to share food and nutrition information. None of the four participants selected Facebook, Twitter, or YouTube as suitable platforms.

### 3.3. Ranking of Mock Instagram Posts

The median rank of mock Instagram posts is shown in Table 2. There was a significant difference between the rank and type of mock Instagram post for engagement (χ^2^ (2) = 63.6, *p* < 0.001), visual preference (χ^2^ (2) = 45.2, *p* < 0.001), motivation (χ^2^ (2) = 61.2, *p* < 0.001), and knowledge relevancy (χ^2^ (2) = 34.1, *p* < 0.001).

The post hoc analysis identified that mock posts in the short video format were consistently rated higher than both the text/icon and realistic image mock post formats. That is, video style posts were reported to be most likely to be engaged with (*p* < 0.001), were visually preferred (*p* < 0.001), were the most motivating to change eating behaviors (*p* < 0.001), and presented the most relevant food and nutrition knowledge (*p* < 0.001) compared to the other post styles. Participants rated that they were more likely to like, share, or save a realistic post than a text/icon equivalent (*p* = 0.007). Realistic posts were also ranked as more motivating than text/icon posts (*p* = 0.016).

The differences in median post rank by participant demographics are presented in Supplementary Table S1. Participant gender, age, level of education, and frequency of Instagram use did not result in a different ranking of video-style posts. However, SES showed differences in video post rank for engagement, motivation to change eating behaviors, and relevancy of food and nutrition knowledge; those of lower SES reported lower rankings. There were some significant differences between education and frequency of Instagram use for text/icon and image-based posts.

## 4. Discussion

This study showed that Instagram was an acceptable and feasible way to deliver nutrition information in this sample. In particular, the participants in this study reported that they preferred video-style posts over both text/icon and realistic image formats.

Young adults are high users of social media [12]. This provides health professionals with a unique chance to engage with this difficult-to-reach population. A systematic review of nutrition interventions delivered through social media to young adults (18–35 years) identified that most studies were conducted using Facebook, Twitter, custom-designed platforms, or applications dedicated to forums [24]. Six-week education programs have been trialed in two different studies conducted in Australians (18–25 years) using Facebook [25] and American student athletes (18–24 years) using Twitter [26]. Both studies found that participants had increased nutrition knowledge at the completion of the intervention [25,26]. However, it has been reported that young adults are moving away from these platforms towards more image- and video-based content such as Instagram [14].

One study conducted in US college students (mean age of 22 years) found that their respondents used Instagram and Snapchat more than Facebook and Twitter [27]. The average number of times Instagram was used per week in the Australian population (>18 years) rose from 29.4 times in 2016 [15] to 37.7 times in 2017 [12]. Meanwhile, over the same period, the average number of times Facebook was used per week declined from 31.9 in 2016 [15] to 25.0 times [12]. Almost all participants in this study reported that Instagram was an acceptable and feasible platform to share nutritional information.

The characteristics of respondents in this pilot study generally mirrored typical Instagram users. In this study, over half of participants reported that they checked Instagram multiple times each day. This is comparable with a previous report that identified that 42% of a sample of US adults visited Instagram multiple times per day [28]. In 2020, 79% of a sample of Australian individuals reported checking their social media accounts at least once per day [29] with 74% of respondents in this study using Instagram a minimum of once per day. In this sample, 45% of respondents were aged 18–24 years, and a similar distribution was reported for Australian Instagram users for September 2022 (46% aged 18–24 years out of users aged 18–34 years) [30]. In a study conducted in the US, 43% of those with a college degree reported using Instagram, and a further 37% of those that indicated having some level of college education also indicated they used this social media platform [31]. In this pilot study, 68% of responders reported being currently enrolled or graduated from a university degree. However, there appears to be an overrepresentation of females and those from higher SES areas in this study, with 56.7% of total Australian Instagram users reporting to be women (77% respondents were female) [30] and only 46–60% of those with an annual income greater than $70,000 USD reporting to use Instagram in 2018 [32].

Post content can take the form of images or videos, and the most suitable post style is dependent on the target population and the participant [33]. Short videos in particular have gained popularity due to their ability to deliver content quickly and concisely [34]. Videos have been reported to be easier to follow than reading text-based content [14]. This is supported by the findings of this study, where young adults ranked video-style posts higher than all other post formats. Previous studies have also reported that cooking videos that targeted barriers to vegetable cooking (aged 18–29 years) [35] and aimed to encourage calcium intake (aged 18–25 years) [36] were well-received by young Australians. Thus, our finding of a higher preference towards video formats within young adults is not unexpected. We also found that realistic image posts were rated higher for engagement when compared to text/icon-based posts. This is similar to previous studies that found that image-based posts had higher levels of engagement when compared to text-based posts when conducted on Facebook in US adults (>35 years) [37] and in a review of posts by Australian public health organizations [38].

One systematic review reported that few studies have attempted to use image-based social media platforms (including Instagram) as an intervention tool for public health [39]. Instagram has the potential to both deliver health promotion campaigns [17,18,40] and encourage adherence to programs [41,42]. The use of short videos in Instagram is even less studied, as the Reels feature was only launched in August 2020 [43]. The Instagram Reels feature (like TikTok) has inbuilt functions that enable users to easily record and edit video content on smartphone devices, reducing requirements for specialized equipment and video editing software. A study conducted using Facebook Live, a real-time online live streaming function, was able extend their reach to deliver nutrition education to lower income Americans (>16 years) [44]. A study conducted in Portuguese adults identified that short videos should be considered when developing mass media health promotion campaigns targeting healthy eating [45]. This was confirmed by a qualitative study conducted in young Australian women (18–35 years) that recommended that effective social media engagement should include engaging content such as videos [46].

To our knowledge, this is the first study that has examined young Australian adult preferences on different post styles using Instagram. However, there are several limitations in this study that reduce its generalizability to young adult populations at large. The use of convenience sampling resulted in the inclusion of a relatively homogenous sample of participants that were predominately female, highly educated, and from higher SES. Differences in volunteers and non-volunteers for research have been previously reported to be female, higher education, and from higher social classes [47]. This is consistent with the finding of one systematic review of online health behavior interventions that reported that on average, 83.3% of participants were female [48]. Individuals with low levels of education and those of lower SES have been previously reported to also be underrepresented in health and medical research [49]. This distribution of participant demographics has been observed in other studies conducted in young Australian adult populations [25,50,51,52]. We did not ask participants about their ethnicity and acknowledge this as a potential unidentified moderator. As this was a pilot study, the target sample size of 100 was deemed to be sufficient to examine the preferred post style of nutritional information for young adults without intention of performing sub-analyses. However, sub-groups may have differential preferences for different post styles. In this study, SES showed differences in the ranking of video style posts. Secondary analysis of the Australian NNPAS 2011–2012 identified sub-groups at risk of consuming high-energy-dense diets, including those with lower education, those born in Australia or an English-speaking country, men with lower income, and women from areas of lower socio-economic status [3]. Future studies should consider recruiting a larger and more diverse sample to examine this further and could employ a recruitment method that enables quotas representative of the target population for gender, ethnicity, and SES factors including level of education and level of income to ensure a diverse range of participants. This will improve the generalizability of findings and enable further sub-group analyses to be performed.

Participants may have been motivated by the monetary incentive (AUD 10 voucher) to respond to the survey. However, as this incentive was low, it would have been unlikely to be a driving factor for participants to complete the survey. Out of all respondents who attempted the screener, 28% did not complete the survey after they became aware that they would not receive an incentive without returning a complete response. We did not examine the reasons for participants withdrawing from survey completion following consent nor the impact of recruitment method on results. Future studies should consider the effects of the method of recruitment into the survey to improve comprehensiveness.

Participants were not asked to rank the post content (only the post style), nor asked to provide feedback on the audio and if captions were required in situations where sound could not be played or heard (e.g., noisy environments). A previous social marketing study investigating market segmentation identified six psycho-behavioral profiles in young adults for healthy lifestyle and food choices [53]. These young adult segments exhibited varied responses to health promotion strategies, and their research highlighted the need to tailor health messages [54]. Future studies should consider employing co-design principles with a panel of diverse young adults in terms of sociodemographic characteristics and young adult market segments to improve the relevance of messages. Co-design requires engaging with end users, health professionals, and other stakeholders to design, implement, and refine end products or services [55]. A systematic review of studies that utilized co-design techniques or participatory research in the development or evaluation of interventions aiming to improve dietary behaviors or nutrition information reported 14 out of 15 interventions had positive health/behavior outcomes [56]. As this survey tested mock posts in an online-survey platform, future studies could also examine the influence of Instagram algorithms as well as using nuanced posts to reach target populations. Algorithms influence how and when posts are displayed to individual users.

## 5. Conclusions

Overall, the Instagram platform was an acceptable and feasible platform to share nutrition information. Video formats were the preferred post style in this sample of young Australian adults. The current survey was limited in size and the sample comprised mostly females of upper socioeconomic status. Further research with a larger and more diverse sample to allow more detailed characterization by sociodemographic and psycho-social variables may be needed to nuance posts to specific target groups. The findings of this pilot study could be used to develop a more tailored approach to the design and testing of nutrition promotions via Instagram for this young adult demographic.

## Figures and Tables

**Table 1:** Instagram survey participant characteristics (*n* = 108).

Characteristic		*n* (%)
Gender	Male	24 (22)
	Female	83 (77)
	“Prefer not to say” and “Other”	1 (1)
Age range (years)	18–24	49 (45)
	25–30	59 (55)
Education	Currently enrolled or graduated from a university degree	73 (68)
	Currently enrolled or graduated from a trade qualification, apprenticeship certificate, or diploma (TAFE or business college)	33 (31)
	School (only)	1 (1)
	Prefer not to say	1 (1)
Socio-economic status (SES) ^1,2^	Lower	10 (11)
	Higher	82 (89)
Frequency of Instagram Use	Multiple times per day	55 (51)
	Once per day	25 (23)
	A few times per week	27 (25)
	Less than once per week	1 (1)

^1^ Socio-economic status (SES) was assigned using the Socio-Economic Indexes for Areas (SEIFA). SEIFA index assigns each area a decile for disadvantage versus advantage with lower deciles demonstrating more disadvantage. The SES category was reduced to the bottom five SEIFA deciles categorized as low SES and top five SEIFA deciles as high SES due to the small sample size in some cells. ^2^ Sixteen participants did not report a valid New South Wales residential postcode and were excluded from the reporting of SES.

**Table 2:** Median (IQR) Rank of Mock Instagram Posts. Friedman test was used to determine if there was a difference between the three post styles. Post hoc analysis conducted using Wilcoxon signed-ranks test with a Bonferroni correction applied to determine differences between the post styles, significance indicated by alphabetical superscripts.

	Median Rank Post (IQR)			*p*-Value
	Text/Icon Only	Realistic Image	Short Video	
Engagement	1.5 (1.0–2.0)	2.0 (1.5–2.0) ^A^	3.0 (2.0–3.0) ^C,D^	<0.001
Visual Preference	2.0 (1.0–2.0)	2.0 (1.0–2.0)	3.0 (2.0–3.0) ^C,D^	<0.001
Motivation	2.0 (1.0–2.0)	2.0 (1.5–2.0) ^B^	3.0 (2.0–3.0) ^C,D^	<0.001
Knowledge Relevancy	2.0 (1.0–2.0)	2.0 (1.5–2.0)	3.0 (2.0–3.0) ^C,D^	<0.001

^A,B^ Statistically significant difference between text/icon only style post compared to realistic image style posts. (A: *p* = 0.007, B: *p* = 0.016). ^C^ Statistically significant difference between text/icon only style post compared to video style post (C: *p* < 0.001). ^D^ Statistically significant difference between realistic image style post compared to video style post. (D: *p* < 0.001).

## Data Availability

Data is available upon request to the authors subject to ethical approval.

## Supplementary Materials

The following supporting information can be downloaded at: https://www.mdpi.com/article/10.3390/nu14204382/s1, Table S1. Median (IQR) Rank of Mock Instagram Posts grouped according to participant demographics (gender, age, education, socio-economic status, and frequency of Instagram use).
