# Supplementary material for: Young Australian Adults Prefer Video Posts for Dissemination of Nutritional Information over the Social Media Platform Instagram: A Pilot Cross-Sectional Survey
Source: Nutrients. 2022 Oct 19;14(20):4382. doi: 10.3390/nu14204382 (PMC9610946; doi:10.3390/nu14204382)
Supplement: Supplementary file 1 [file nutrients-14-04382-s001.zip › nutrients-1932372-supplementary.pdf]

Table S1. Median (IQR) Rank of Mock Instagram Posts grouped according to participant demographics (gender, age, education, socio-economic status and frequency of Instagram use). Mann-Whitney U tests used to determine if there was a difference between participant characteristics and post rank with a Bonferroni correction applied. Significance indicated by alphabetical superscripts.

	**Median Rank Post (IQR)**					
	Text/Icon Only		Realistic Image		Short Video	
**Gender**	Male	Female	Male	Female	Male	Female
(n)	24	83	24	83	24	83
Engagement	1.5 (1.0 – 2.0)	1.5 (1 - 2.0)	2.0 (2.0 - 2.8)	2.0 (1.0 - 2.0) [A]	2.5 (2.0 - 3.0)	3.0 (2.0 - 3.0)
Visual Preference	1.8 (1.0 - 2.3)	2.0 (1.5 - 2.0)	2.0 (2.0 - 2.3)	2.0 (1.0 - 2.0)	2.0 (2.0 - 3.0)	3.0 (2.0 - 3.0)
Motivation	1.5 (1.0 - 2.0)	2.0 (1.0 - 2.0)	2.0 (2.0 - 2.0)	2.0 (1.0 - 2.0)	2.5 (2.0 - 3.0)	3.0 (2.0 - 3.0)
Knowledge Relevancy	1.5 (1.0 - 2.0)	2 (1.0 - 2.0)	2.0 (1.8 - 3.0)	2.0 (1.0 - 2.0)	2.0 (2.0 - 2.5)	3.0 (2.0 - 3.0)
**Age-range**	18-24	25-30	18-24	25-30	18-24	25-30
(n)	49	59	49	59	49	59
Engagement	2.0 (1.0 - 2.0)	1.5 (1.0 - 2.0)	2.0 (1.5 - 2.0)	2.0 (1.5 - 2.0)	3.0 (2.0 - 3.0)	3.0 (2.5 - 3.0)
Visual Preference	2.0 (1.0 - 2.0)	2.0 (1.0 - 2.0)	2.0 (1.0 - 2.0)	2.0 (1.0 - 2.0)	3.0 (2.0 - 3.0)	3.0 (2.0 - 3.0)
Motivation	2.0 (1.0 - 2.0)	1.5 (1.0 - 2.0)	2.0 (1.5 - 2.0)	2.0 (1.5 - 2.0)	3.0 (2.0 - 3.0)	3.0 (2.0 - 3.0)
Knowledge Relevancy	2.0 (1.0 - 2.0)	2.0 (1.0 - 2.0)	2.0 (1.0 - 2.0)	2.0 (1.5 - 2.0)	2.5 (1.5 - 3.0)	3.0 (2.0 - 3.0)
**Education**	University	All other education	University	All other education	University	All other education
(n)	73	35	73	35	73	35
Engagement	1.5 (1.0 - 2.0)	2.0 (1.5 - 2.0) [B]	2.0 (2.0 - 2.0)	1.5 (1.0 - 2.0) [C]	3.0 (2.0 - 3.0)	3.0 (2.5 - 3.0)
Visual Preference	2.0 (1.0 - 2.0)	2.0 (2.0 - 2.0) [C]	2.0 (1.5 - 2.5)	1.0 (1.0 - 2.0) [C]	3.0 (2.0 - 3.0)	3.0 (2.0 - 3.0)
Motivation	1.5 (1.0 - 2.0)	2.0 (1.5 - 2.0) [D]	2.0 (2.0 - 2.0)	1.5 (1.0 - 2.0) [E]	3.0 (2.0 - 3.0)	3.0 (2.5 - 3.0)
Knowledge Relevancy	1.5 (1.0 - 2.0)	2.0 (2.0 - 2.0)	2.0 (2.0 - 2.5)	1.5 (1.0 - 2.0) [C]	2.5 (2.0 - 3.0)	3.0 (2.0 - 3.0)
**Socio-economic status**	Higher	Lower	Higher	Lower	Higher	Lower
(n)	82	10	82	10	82	10
Engagement	1.5 (1.0 - 2.0)	2.0 (1.0 - 2.0)	2.0 (1.5 - 2.0)	2.0 (2.0 - 3.0)	3.0 (2.5 - 3.0)	2.0 (1.0 - 2.5) [F]
Visual Preference	2.0 (1.0 - 2.0)	2.0 (2.0 - 2.0)	2.0 (1.0 - 2.0)	2.0 (2.0 - 3.0)	3.0 (2.5 - 3.0)	2.3 (1.0 - 3.0)
Motivation	1.5 (1.0 - 2.0)	2.0 (2.0 - 2.0)	2.0 (1.5 - 2.0)	2.0 (2.0 - 3.0)	3.0 (2.5 - 3.0)	2.0 (1.0 - 2.5) [F]
Knowledge Relevancy	2.0 (1.0 - 2.0)	2.0 (1.0 - 2.0)	2.0 (1.0 - 2.0)	2.0 (2.0 - 2.5)	3.0 (2.0 - 3.0)	2.0 (1.0 - 2.5) [G]
**Frequency of Instagram Use**	Multiple times per day	Once per day or less	Multiple times per day	Once per day or less	Multiple times per day	Once per day or less
(n)	55	53	55	53	55	53
Engagement	1.0 (1.0 - 2.0)	2.0 (1.5 - 2.0) [H]	2.0 (2.0 - 2.0)	2.0 (1.0 - 2.0) [K]	3.0 (2.0 - 3.0)	3.0 (2.0 - 3.0)
Visual Preference	1.5 (1.0 - 2.0)	2.0 (2.0 - 2.0) [I]	2.0 (2.0 - 2.5)	1.5 (1.0 - 2.0) [J]	2.5 (2.0 - 3.0)	3.0 (2.5 - 3.0)

Motivation	1.0 (1.0 - 2.0)	2.0 (1.5 - 2.0) [J]	2.0 (2.0 - 2.5)	1.5 (1.0 - 2.0) [J]	3.0 (2.0 - 3.0)	3.0 (2.0 - 3.0)
Knowledge Relevancy	1.5 (1.0 - 2.0)	2.0 (2.0 - 2.0) [J]	2.0 (2.0 - 2.5)	1.5 (1.0 - 2.0) [J]	2.5 (2.0 - 3.0)	3.0 (2.0 - 3.0)

[A] Statistically significant difference between males and females. (A: $p = 0.013$)

[B, C., D, E] Statistically significant difference between university and all other education (B: $p = 0.009$, C: $p < 0.001$, D: $p = 0.001$, E: $p = 0.002$)

[F, G] Statistically significant difference between higher and lower socio-economic status (F: $p = 0.001$, G: $p = 0.004$)

[H, I, J, K] Statistically significant difference between using Instagram multiple times per day and once per day or less (H: $p = 0.002$, I: $p = 0.001$, J: $p < 0.001$, K: $p = 0.013$)
